# Comparison of the retroperitoneal versus Transperitoneal laparoscopic Adrenalectomy perioperative outcomes and safety for Pheochromocytoma: a meta-analysis

**DOI:** 10.1186/s12893-020-0676-4

**Published:** 2020-01-13

**Authors:** Yu-Li Jiang, Lu-Jie Qian, Zhen Li, Kang-Er Wang, Xie-Lai Zhou, Jin Zhou, Chun-Hua Ye

**Affiliations:** 1 Department of Urology, The Affiliated Hospital of Hang Zhou Normal University, Hangzhou, 310015 China; 2 School of Medicine, Hang Zhou Normal University, Hangzhou, 310016 China

**Keywords:** Retroperitoneal, Transperitoneal, Laparoscopic, Adrenalectomy, Pheochromocytoma, Meta-analysis

## Abstract

**Background:**

To compare the perioperative outcomes and safety of transperitoneal laparoscopic adrenalectomy with those of retroperitoneal laparoscopic adrenalectomy for patients with pheochromocytoma.

**Methods:**

We searched PubMed, EMBASE and the Cochrane Central Register for studies from 1999 to 2019 to assess the perioperative outcomes and safety of transperitoneal laparoscopic adrenalectomy and the retroperitoneal approach for laparoscopic adrenalectomy in patients with pheochromocytoma. After data extraction and quality assessments, we used RevMan 5.2 to pool the data.

**Results:**

Four retrospective studies were obtained in our meta-analysis. Patients who underwent retroperitoneal laparoscopic adrenalectomy were associated with shorter operative time (WMD: 34.91, 95% CI: 27.02 to 42.80, I2 = 15%; *p* < 0.01), less intraoperative blood loss (WMD: 139.32, 95% CI: 125.38 to 153.26, I2 = 0, *p* < 0.01), and a shorter hospital stay (WMD: 2, 95% CI: 1.18 to 2.82, I2 = 82%, *p* < 0.01) than patients who underwent transperitoneal laparoscopic adrenalectomy. No significant differences were found in the complication rate (OR: 1.58, 95% CI: 0.58 to 4.33, I^2^ = 0; *p* = 0.38) or in the incidence of hemodynamic crisis (OR: 0.74, 95% CI: 0.19 to 2.94, *p* = 0.67) between the two groups.

**Conclusion:**

Retroperitoneal laparoscopic adrenalectomy could achieve better perioperative outcomes than the transperitoneal approach for patients with pheochromocytoma.

## Background

Pheochromocytoma (PHEO) is composed of chromaffin cells that arise from the extra-adrenal paraganglia or adrenal medulla. Patients with PHEO usually present with symptoms attributable to hormonal secretion or an incidentally detected mass on imaging studies [1]. Unregulated excessive catecholamine secretion can lead to myocardial infarction, acute respiratory distress syndrome, cerebrovascular accidents and acute renal failure [1, 2]. The widespread availability of ultrasound and computed tomography allows for increased incidental detection of PHEOs.

Surgical resection is the treatment of choice for pheochromocytoma. Gagner performed the first laparoscopic adrenalectomy [3]. Starting in the past decade, minimally invasive adrenalectomy is now considered a gold standard for the treatment of adrenal tumors [4]. Several studies have reported that no significant difference was found in operative time, estimated blood loss, or complication rates between the transperitoneal laparoscopic adrenalectomy (TLA) and posterior retroperitoneal laparoscopic adrenalectomy (PRA) approaches [5–7]. TLA is the preferred technique of many surgeons due to the familiar intraperitoneal anatomy and large working space [8]. The posterior retroperitoneoscopic approach provides good visualization of the retroperitoneal anatomy, direct access to the adrenal tumor without mobilization of visceral organs while maintaining high insufflation pressures to establish an operating space and acquiring fewer blood loss by tamponading small bleeding vessels, making it convenient to remove adrenal tumors.

Several studies have been conducted to compare both surgical approaches. Several studies have shown that the outcomes of PRA were superior to TLA for removing adrenal tumors in terms of less blood loss and shorter operation time [9–11]. However, there is a lack of data regarding the comparison of the two approaches in patients with PHEO.

Three meta-analyses were performed to compare laparoscopic retroperitoneal versus transperitoneal adrenalectomy for adrenal tumors [12–14]. Nigri et al. performed a meta-analysis comparing TLA versus PRA to treat adrenal tumors and indicated that no significant differences existed between the two surgical procedures [12]. Chen et al. conducted a meta-analysis reported that patients in the PRA group could achieve better clinical outcomes than the patients in the TLA group [13]. Constantinides et al. performed a meta-analysis found that patients who underwent PRA acquired equivalent outcomes to patients who underwent TLA, except for hospital stay [14]. However, no meta-analysis has been conducted to evaluate the two surgical methods.

The aim of this study was to compare the perioperative clinical outcomes and safety of retroperitoneal versus transperitoneal for laparoscopic adrenalectomy in patients with pheochromocytoma.

## Methods

### Search strategy

We performed this meta-analysis following the Preferred Reporting Items for Systematic Reviews and Meta-Analysis (PRISMA) guidelines [15]. We searched PubMed, EMBASE and the Cochrane Central Register for relevant studies published in English between 1999 and 2019. We used the following search terms: “laparoscopy [MeSH]”, “pheochromocytoma [MeSH]”, “adrenalectom* OR pheochromocytoma [MeSH]”, and “retroperiton* OR transperiton* OR laparoscop*”. We also used the combined Boolean operators “AND” or “OR” in the title/abstract.

### Inclusion and exclusion criteria

The inclusion criteria were as follows: (1) comparative study of TLA and PRA for the treatment of PHEOs; (2) studies that contained at least one of the following outcomes: operative time, intraoperative blood loss, length of hospital stay, complication rate, and incidence of hemodynamic instability crisis; (3) comparative studies of the retroperitoneal and transperitoneal approaches. Two investigators (YLJ and LZ) reviewed the articles.

**Fig. 1:**
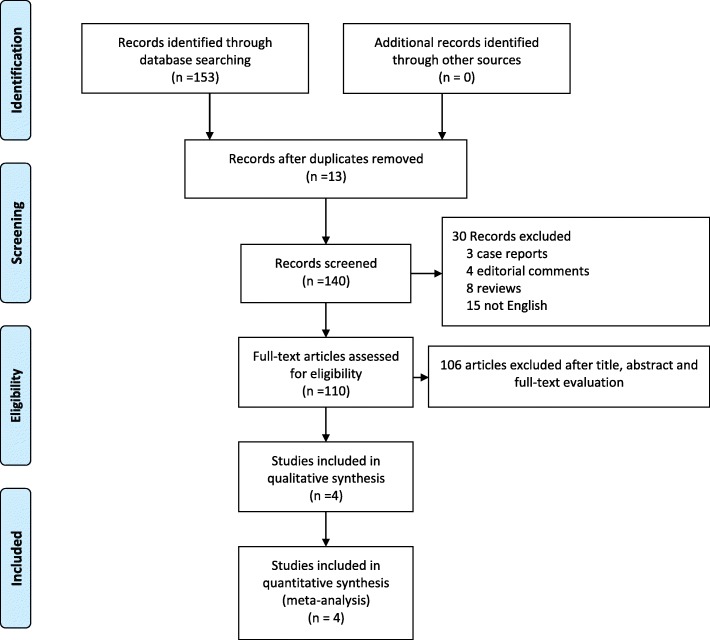
Flow diagram of the process for the selection of relevant studies

The exclusion criteria were as follows: (1) case reports, editorial comments, meeting abstracts, reviews and articles without applicable data; (2) studies with insufficient data, such as those that lacked means and standard deviations; and (3) studies that were single-arm trials or not comparative. The process of identifying relevant studies is summarized in Fig. 1.

### Data extraction

These two authors extracted data, such as the operation time, blood loss, length of hospital stay, complication rate, conversion rate and incidence of hemodynamic crisis. The following data were recorded: (1) baseline comparative data: study year, design, sample size, mean age, body mass index, and tumor size; (2) intraoperative clinical outcomes: operative time, blood loss, number of complications, conversion rate, length of hospital stay; (3) postoperative complications. Any disagreements were resolved by discussion.

### Statistical analysis

We used Review Manager Version 5.2 software (The Cochrane Collaboration, Oxford, UK) to perform the analysis of the included data. We evaluate the heterogeneity with Cochran’s Q; if the value of Q < 50% or *P* > 0.01, we believed little heterogeneity was existed. However, if Q > 50% and *P* < 0.01, evident heterogeneity existed. If I^2^ > 50%, the random effects model was applied. For quantitative data, we used weight mean difference (WMD) or standard mean difference (SMD) to calculate continuous data. We used the OR and 95% confidence interval (CI) to evaluate binary data. All tests were 2-tailed, and the statistical significance level was set at 0.05.

## Results

### Literature search

Four studies were included in our study [16–19]. The PRISMA flow diagram of the search process is summarized in Fig. 1. From the selected databases, our search obtained 153 reports. We removed 13 duplicates. After screening the titles and abstracts, 30 full texts were excluded, of which, 15 reports were not in English, 8 reports were reviews, 4 reports were editorial comments and 3 reports were case reports. The remaining 110 reports underwent a comprehensive and detailed evaluation. Ultimately, 4 studies were included in this meta-analysis. Table 1 summarizes the baseline characteristics and assessments of the included studies.

**Table 1:** Basic characteristics of the included studies

Study	Year	Design	Sample Size		Tumor Size (cm)		BMI	
			TLA	PRA	TLA	PRA	TLA	PRA
Li	2010	R	40	59	4.5	4.3	25.5	26.31
Dickson	2011	R	23	23	4.0	3.3	26.1	26.2
Kiriakopoul	2015	R	17	19	5.1	3.7	NA	NA
Shiraishi	2019	R	12	10	6.6	7.4	23.4	23.2

*R* retrospective study, *NA* not available, *TLA* transperitoneal laparoscopic adrenalectomy, *PRA* posterior retroperitoneal laparoscopic adrenalectomy, *BMI* body mass index

### Quality assessment

We used the New-Ottawa Scale (NOS) to assess the included nonrandomized studies. We used a 9-point system to evaluate the NOS scores. The study score of 7–9 or above was considered high quality, a score of 4–6 was considered medium quality, and a score of 0–4 or below was considered low quality. Two reviewers (YLJ and LJQ) evaluate the quality of the included studies. Table 2 presents the quality assessments of the included studies.

**Table 2:** Quality assessment of the included studies

Study	Design	Selection				Comparability	Outcome			Total
		Representativeness of exposed cohort	Selective of nonexposed Cohort	Ascertainment of exposure	Outcome not present at start		Assessment of outcome	Adequate follow-up length	Adequacy of follow-up	
Li	R	*		*	*	**	*	*	*	8
Dickson	R	*		*	*	**	*	*	*	8
Kiriakopoul	R	*			*	**	*	*	*	7
Shiraishi	R	*		*	*	**	*	*	*	8

*R* Retrospective study The symbol “*” represents score

### Operation time

The operation time was investigated in two studies. A total of 145 patients were included (63 in the TLA group and 82 patients in the PRA group) for analysis.

**Fig. 2:**
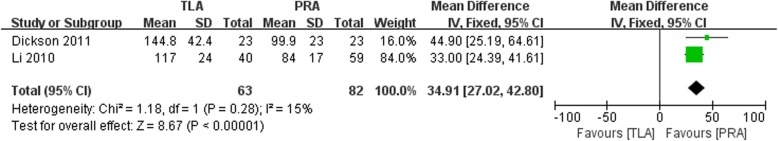
Forest plot for the operation time between the TLA and PRA for PHEO

The operation time was longer in the TLA group than in the RPA group (WMD: 34.91, 95% CI: 27.02 to 42.80, I^2^ = 15%; *p* < 0.01, fixed-effects model, Fig. 2).

### Blood loss

**Fig. 3:**
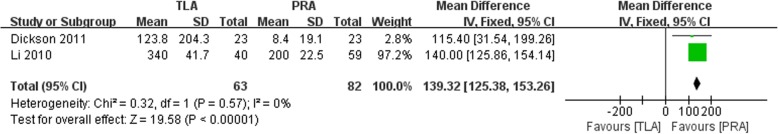
Forest plot for the blood loss between the TLA and PRA for PHEO

We acquire the relevant data of blood loss from two studies. This study showed that patients in the TLA group lost significantly more blood than those in the PRA group (WMD: 139.32, 95% CI: 125.38 to 153.26, I^2^ = 0, *p* < 0.01, fixed-effects model, Fig. 3).

### Length of hospital stay

**Fig. 4:**
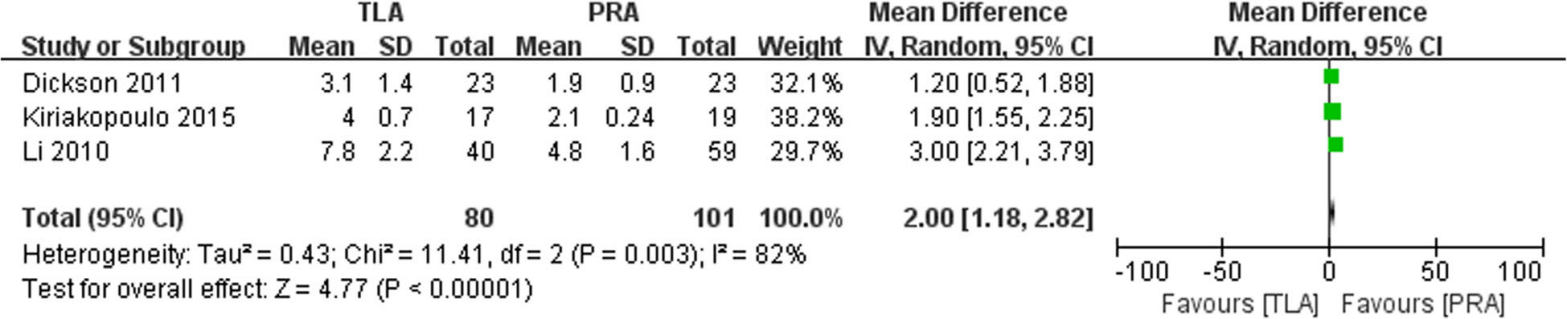
Forest plot for the hospital stay between the TLA and PRA for PHEO

Three studies reported the length of hospital stay. Significant heterogeneity was existed in the pooled data. The pooled data indicated that patients in the TLA group stayed significantly longer than those in the PRA group (WMD: 2, 95% CI: 1.18 to 2.82, I^2^ = 82%, *p* < 0.01, random-effects model, Fig. 4).

### Complication rate

**Fig. 5:**
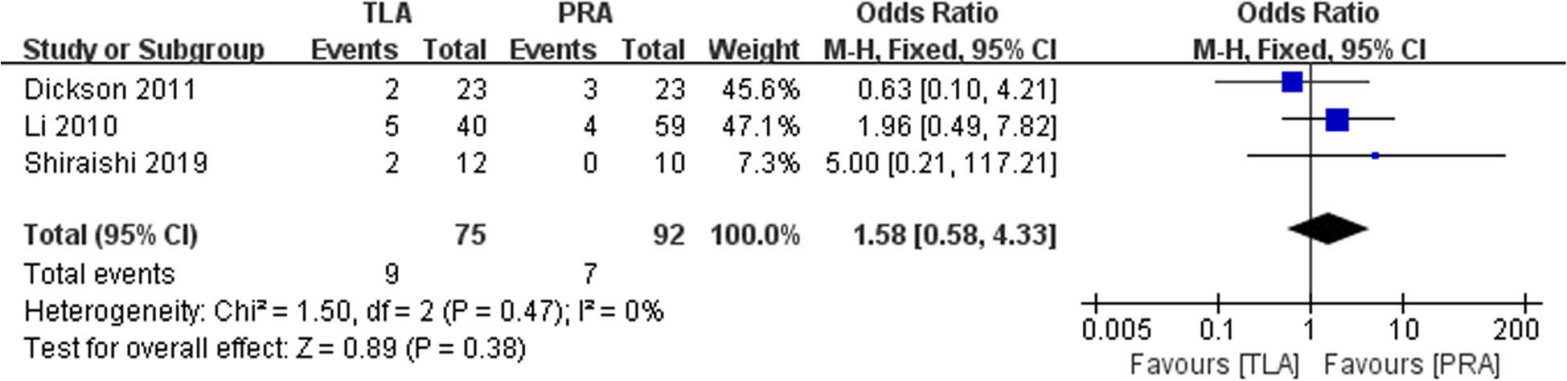
Forest plot for the complication between the TLA and PRA for PHEO

Three trials, which included a total of 167 patients, were included in the meta-analysis to evaluate the perioperative complication rate. No significant difference between the TLA and PRA groups was noted (OR: 1.58, 95% CI: 0.58 to 4.33, I^2^ = 0; *p* = 0.38, fixed-effects model, Fig. 5).

### Conversion rate

**Fig. 6:**
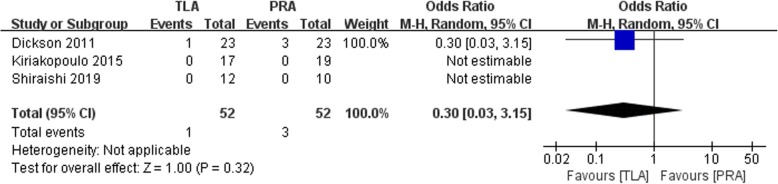
Forest plot for the conversion between the TLA and PRA for PHEO

Data related to conversion rate were obtained in three studies. No significant difference between the TLA and PRA groups was found (OR: 0.30, 95% CI: 0.03 to 3.15, *p* = 0.32, random-effects model, Fig. 6).

### Hemodynamic crisis

**Fig. 7:**
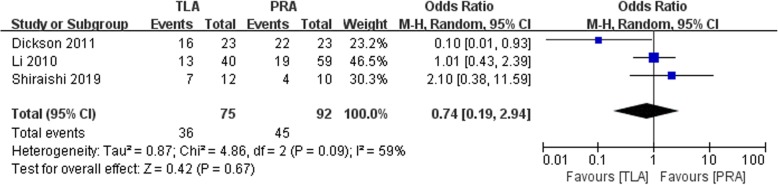
Forest plot for the Hemodynamic crisis between the TLA and PRA for PHEO

Among the three included studies reporting the operative incidence of hemodynamic crisis, no significant difference between the TLA and PRA groups was found (OR: 0.74, 95% CI: 0.19 to 2.94, *p* = 0.67, random-effects model, Fig. 7).

## Discussion

In our meta-analysis, we included four retrospective studies and analyzed the two surgical approaches for the treatment of pheochromocytoma. This meta-analysis is the first meta-analysis to compare transperitoneal versus retroperitoneal laparoscopic adrenalectomy for pheochromocytoma. In our study, the PRA group achieved better outcomes in terms of operation time, postoperative length of hospital stay and blood loss than the TLA group (Fig. 4). No significant differences were found between the two groups in complication rate, conversion rate and incidence of hemodynamic crisis.

In our meta-analysis, the patients in the TLA group had a longer operation time than in the PRA group (*p* < 0.01). Kiriakopoulos et al. conducted a retrospective study that contained 34 patients and reported a similar result (*p* = 0.007). They also performed a pearson analysis to explore the relationship between the operative time and the baseline tumor size and age of the patient (*p* < 0.05) [18]. Chen et al. performed a meta-analysis consisting of 9 retrospective studies which included patients with PHEO, aldosteronoma (ALD), Cushing syndrome (Cu) and malignant tumors such as adrenal cortical carcinoma (ACC). They concluded that the operation time was significantly shorter in the PRA group than in the TLA group (WMD: 13.10, 95% CI: 23.83 to − 2.36, *p* < 0.05) [13]. This result may be attributed to the easy establishment of the pneumoperitoneum and fewer trocars needed for PRA than for TLA. In addition, surgeons could dissect the PHEO directly by theTLA, which was superior to the PRA. However, Chiang et al. included 55 patients with right-sided benign tumors in a single center (31 in the PRA group and 24 in the TLA group) and indicated that no statistically significant difference was available between the two groups (*p* = 0.471) [20]. They also involved patients with different pathological tumors (PHEO, Conn syndrome, Cushing syndrome, nonfunctioning adenoma, etc.), which may have led to the contrary conclusion to that in our study.

Several studies have reported that the transperitoneal approach was better for the managements of large adrenal masses (> 5 cm) with shorter operative time and good exposure of the anatomic landmarks compared to the retroperitoneal approach [9, 17, 21].

This study indicated that patients in the TLA group had significantly larger tumors than those in the PRA group (*n* = 145, WMD: 139.32, 95% CI: 125.38 to 153.26, I^2^ = 0, *p* < 0.01). Nigri et al. conducted a meta-analysis consisting of 21 studies which included both retrospective, randomized control trials and case-control studies. They reported that no significant difference was found between the two groups (*p* = 0.15) [12]. This result was not consistent with our study. They included studies which recruited both patients with benign and malignant tumors. In their study, the different tumor characteristics may add heterogeneity to the study. Shiraishi et al. included 22 patients with large pheochromocytomas (mean tumor size > 7 cm) and showed a similar result to that of our meta-analysis. Kiriakopoulos also demonstrated that the dissection and the ligation of the right adrenal vein could provide an easy control of the right adrenal vein in the PRA approach [18]. Li et al. performed a retrospective study which involved 99 patients with pheochromocytomas comparing TLA with PRA and stated that the mean operative time (117 vs 84 min) was significantly longer in the TLA group than in the PRA group, infering a similar result to our study.

Dickson et al. performed a study that included 46 patients with PHEOs. There were no differences in age, BMI, or tumor size between these groups. A significant reduction in blood loss was observed in patients who in PRA compared to those who in TLA (*p* = 0.02). The difference is likely due to the difficulty to expose the adrenal gland, as well as the high retroperitoneal insufflation pressures, which tamponade bleeding from small vessels [17].

In our study, we found that patients in the TLA group stayed significantly longer in the hospital than those in the PRA group (*p* < 0.01, Fig. 4). However, Chai et al. reported that no significant difference was existed between the TLA and PRA groups (*p* = 0.558) [9]. Dickson et al. and other studies also reported similar results. This is because the patients in the PRA group have a lower chance of bowel mobilization and a faster rate of tolerating a general diet by the next morning after surgery than patients in the TLA group. Similarly, Chen et al. conducted a meta-analysis in 2013 and found that the length of hospital stay was shorter in the PRA group than in the TLA group (WMD: 1.25; 95% CI: − 2.36 to − 0.14; *p* = 0.03) [13]. However, another meta-analysis conducted by Nigri et al. indicated that no significant difference was found between the groups (*p* = 0.09) [12]. This difference was due to the different inclusion criteria. Compared to the study from Nigri et al., our study included only four studies, which could increase bias.

Our study reported no significant difference between the TLA and PRA groups in terms of complication rate (OR: 1.58, 95% CI: 0.58 to 4.33, *p* > 0.05, Fig. 5). Similarly, a recent meta-analysis of all adrenal tumors showed that no significant difference was found between the two surgical approaches [12]. The authors found that the rates of both major and minor complications were comparable between the two groups (*p* > 0.05). Additionally, Chen et al. also performed a meta-analysis and reported a similar result (OR: 0.53; 95% CI: 0.17 to 1.60; *p* = 0.26) [13].

In the present study, no significant difference between the TLA and RPA groups of conversion rate was found (OR: 0.30, 95% CI: 0.03 to 3.15, *p* = 0.32, Fig. 6). Dickson et al. published a retrospective study that reported conversion to an open procedure in 1 patient in the TLA group and 3 patients in the PRA group [17]. This may be due to the highly vascular nature of PHEOs, which are more technically challenging to remove via PRA than other adrenal tumors. The PRA surgical procedure is more technically demanding since it has a smaller working space restricted to the retroperitoneum than the traditional laparoscopic approach. Kercher et al. conducted a retrospective study that included 81 patients with pheochromocytomas who underwent laparoscopic resection and indicated no difference in conversions between large (> 6 cm) and small tumors (< 6 cm) [22]. This outcome is consistent with the results of our study.

In our study, no significant difference between the TLA and PRA groups was found in terms of the incidence of hemodynamic crisis (OR: 0.74, 95% CI: 0.19 to 2.94, *p* = 0.67, Fig. 7). Shiraishi et al. reported a retrospective study that found that TLA had a higher incidence of hypertensive crisis than PRA (58% vs 40%, retrospectively) [19]. Wessel et al. performed a retrospective, multicenter study that included 341 patients and found that the overall and cardiovascular morbidity rates were comparable between the two approaches [23]. After a multivariate analysis, they found that the two operative approaches did not affect operative hemodynamic instability. PRA increased the incidence of mean arterial pressure < 60 mmHg (OR: 6.255, 95% CI: 1.134–34.235, *p* = 0.035) compared with transperitoneal adrenalectomy by multivariate analysis [23].

Our study was restricted by several limitations. First, the included studies were not RCTs. This can lower the confidence of our study. Second, the inclusion criteria did not involve two-sided PHEOs. The presence of unilateral or two-sided PHEOs could make a difference in selecting the surgical approach. Additionally, the variable tumor size also contributes to the data heterogeneity. We did not include postoperative oncological outcomes or adjust the common baseline characteristics of the patients (tumor size, BMI). For example, obese patients could require a more difficult operation process than nonobese patients. Third, the low number of patients included could have limited the statistical power [12]. The few included studies restricted the ability to perform sensitivity analysis and publication bias analysis to explore the potential data heterogeneity.

## Conclusion

In our meta-analysis, PRA was superior to TLA in clinical efficiency improvements for patients with pheochromocytoma. More multicenter, high-quality RCTs with large sample sizes are needed to verify the perioperative outcomes and safety of TLA and PRA for patients with pheochromocytoma.

## Data Availability

All data generated or analyzed during this study are included in this published article.

## Abbreviations

BMI: Body mass index
CI: Confidence interval
OR: Odds ratio
PHEO: Pheochromocytoma
PRA: Posterior retroperitoneal laparoscopic adrenalectomy
TLA: Transperitoneal laparoscopic adrenalectomy
WMD: Weight mean difference
